# Functional estrogenic activity in rivers, water supply, and rainwater

**DOI:** 10.1016/j.isci.2026.116372

**Published:** 2026-06-18

**Authors:** Gihani Manodara, Ashley Gillon, Emma S. Sutherland, Kelsey L. Stevens, Caitlin M. Deans, Alexia Kauff, Alison K. Heather

**Affiliations:** 1 Department of Physiology, School of Biomedical Science, University of Otago, Dunedin, New Zealand; 2 Otago Medical School, University of Otago, Dunedin, New Zealand; 3 InsituGen Limited, University of Otago, Dunedin, New Zealand; 4 Commercial Research & Development, ALS Food and Environmental NZ, Ruakura Campus, Hamilton, New Zealand

**Keywords:** Environmental chemistry, Biochemistry, Biochemistry methods, Ecological biochemistry

## Abstract

Estrogenic endocrine-disrupting chemicals (eEDCs) are a significant concern due to their widespread contamination of water sources and their potential to disrupt both human and ecological health. The assessment of EDC contamination focuses on detecting only targeted compounds and as a result, the true extent of the risk remains unknown. This study describes the development and application of a novel *in vitro* transcription (IVT)-based reporter gene assay for detecting estrogenic activity from natural-, drinking-, tank-, rain-, and bottled-water. Using estradiol (E2)-spiked waters, the assay demonstrated high sensitivity with a detection limit of 1.0 pg/mL. When applied to the different water sources tested, the results revealed estrogenic activity across most of them. This study shows the need for improved monitoring tools that can detect estrogenic effects of complex EDC mixtures. Employing such tools provides a more comprehensive view of contamination revealing broader human and environmental risks posed by EDCs.

## Highlights

• Rapid cell-free ER transcription bioassay detects estrogenic activity in water
• Bioassay measures cumulative estrogenic activity, capturing mixture effects
• Estrogenic activity was detected in surface waters and drinking water sources
• Sensitive screening tool detects biologically relevant estrogenic activity

## Graphical abstract

**Figure:**
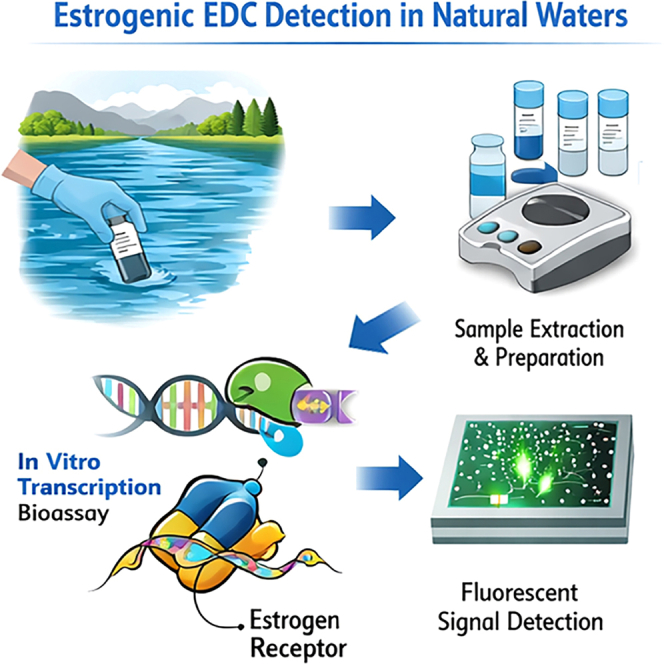

## Introduction

For many regions across the world, water quality is increasingly becoming a significant concern due to contamination with endocrine-disrupting chemicals (EDCs) and other pollutants. The World Health Organization (WHO) defines EDCs as substances that can alter endocrine system function and cause adverse health effects.^1^ Among EDCs, compounds with estrogenic activity are of particular importance because of their ability to mimic or antagonize natural estrogens, potentially leading to deleterious health and ecological impacts. Estrogenic EDCs (eEDCs) including endogenous and synthetic estrogens, disinfection byproducts, fluorinated substances, bisphenols, phthalates, pesticides, and fertilizers have all been identified as major water contaminants.^2^ Natural waters, including rivers, creeks, and lakes, are vulnerable to contamination by eEDCs due to their continuous exposure to agricultural runoff, industrial effluents, wastewater discharge, and urban stormwater.^3^^,^^4^ Even at low concentrations in the range of parts per trillion, eEDCs can disrupt the reproductive biology of aquatic organisms.^5^ In fish, eEDC exposure can feminize males completely, or disrupt male reproductive capacity by driving the production of the female egg protein, vitellogenin.^6^ In amphibians and reptiles, eEDCs alter sex ratios toward females and impair fertility.^7^

eEDCs are associated with the development of various endocrine disorders in humans. Reported conditions include reduced sperm count, endometriosis, early puberty, polycystic ovary syndrome, thyroid dysfunction, diabetes, obesity, and cancers of the breast, testes, and prostate.^8^^,^^9^ Notably eEDCs have a negative impact on embryonic development and placental health. For example, exposure to eEDCs during critical periods of development can result in reduced embryo numbers and impaired implantation processes in female rats.^10^^,^^11^ This may be due to epigenetic changes, such as altered DNA methylation patterns in placental tissues^12^ which are linked to adverse birth outcomes, including low birth weight and developmental abnormalities. eEDCs are also reported to interfere with maternal-fetal exchange processes, affecting nutrient delivery and waste removal during pregnancy.^13^ These findings are consistent with studies, which show that prenatal exposure to eEDCs is associated with an increased risk of being born small for gestational age and/or preterm delivery.^14^ Moreover, eEDCs have the ability to imprint disease pathways, causing long-lasting effects. Subsequent generations may also exhibit issues, particularly in relation to altered metabolic pathways. For instance, exposure to phthalates has been shown to affect sperm quality and reproductive function in F1 to F4 offspring, demonstrating that the effects of eEDCs effects can persist through several generations.^15^

At present, LC-MS/MS and GC-MS are the gold-standard methodologies for detecting eEDCs and have been very effective at determining the presence of estrogenic compounds in aquatic environments.^16^^,^^17^ Although these approaches are exquisitely sensitive, they are designed to detect a small number of specific eEDCs individually. These eEDCs have been identified previously as being potentially harmful to human health based on toxicological studies and/or their persistence in the environment.^18^ Bioassays provide a complementary approach to chemical analysis by measuring the net estrogenic activity of a water sample.^19^ This offers a more holistic assessment of the potential estrogenic effects and, consequently, the biological impacts of eEDCs. This approach is particularly relevant to water testing, where complex eEDC mixtures are commonly present. The most practical bioassays for detecting estrogenic activity are cell-based assays that employ a reporter gene output, as they integrate the effects of all estrogenic substances in the sample, including unknown compounds that may not be detectable through targeted chemical approaches.^20^ The ability to measure biological effects is critical for environmental monitoring, as low concentrations of diverse eEDCs, many of which can act additively or synergistically, can be detected through a significant biological response. Cell-based bioassays are effective for detecting estrogens and have been widely used for detecting contaminated water supplies for eEDCs. However, despite their sensitivity, they require sophisticated equipment and complex protocols to be followed by specialized personnel that make them unsuitable for routine or high-throughput testing. These limitations primarily restrict their broader application outside of dedicated research settings.

We present a newly developed cell-free, *in vitro* transcription-based reporter gene assay for detecting estrogenic activity in water samples. This assay offers a streamlined, easy-to-use format that removes the need for live cells and specialized equipment, addressing key limitations of traditional cell-based systems. Its simplicity and robustness make it well-suited for routine and high-throughput screening in both research and applied testing environments. We have applied this assay to measure estrogenic bioactivity across a diverse array of water sources, including natural waterways, springs, tank and rainwater, and drinking water from taps, fountains and bottles.

## Results

**Figure 1:**
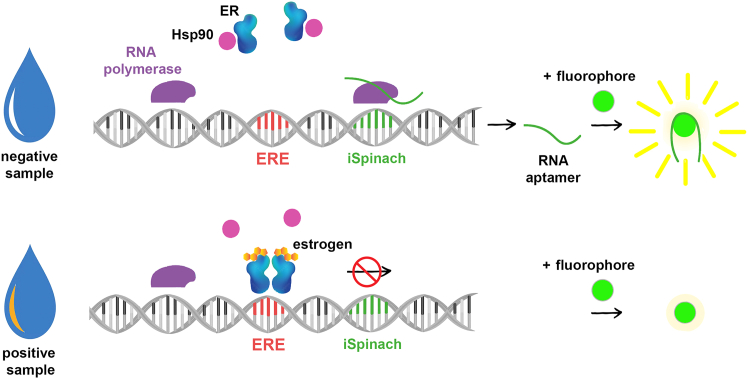
Schematic of the cell-free estrogen bioactivity assay Each reaction is assembled with a DNA template harboring an estrogen response element (ERE, red), estrogen receptor (ER, blue) lysate and HSP90 protein (pink circle), T7 RNA polymerase (purple), and a reaction mix to support *in vitro* transcription (IVT). The IVT reactions generate iSpinach RNA aptamer (dark green) that is detected by binding specifically to the DFHBI-1T fluorophore (light green circle). For an estrogen-negative sample, T7 RNA polymerase transcribes the DNA template and produces iSpinach RNA aptamer. iSpinach binds to DFHBI-1T and fluorescence is measured. ER remains in an inactive state, inhibited by bound HSP90. For a positive sample, estrogens activate ER, triggering a conformational changed that releases HSP90 and exposes a DNA binding site, allowing ER to bind to the ERE. Once bound to the DNA template, ER sterically inhibits T7 RNA polymerase from transcribing the DNA template. Thus, no iSpinach RNA aptamer is produced and there is no binding to DFHBI-1T to produce fluorescence. DFHBI-1T= (Z)-5-(3,5-difluoro-4-hydroxybenzylidene)-2-methyl-3-(2,2,2-trifluoroethyl)-3,5-dihydro-4H-imidazol-4-one.

**Figure 2:**
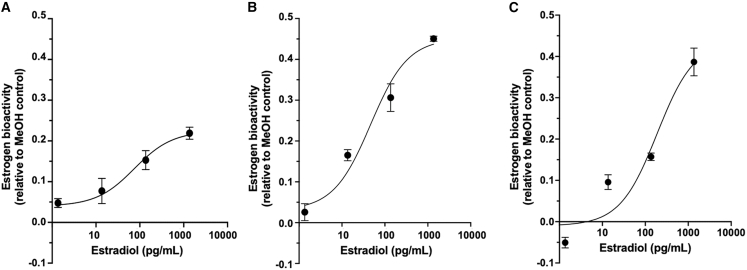
Dose-response curves for estradiol Representative 17β-estradiol dose-response curves in which the ligand solubilized in 50% (A) or 10% (B) methanol vehicle was added directly to the ER-IVT reaction tubes or was spiked into laboratory grade MilliQ water and SPE column extracted (C) before dried eluate was reconstituted in 10% methanol vehicle before testing. Data are shown as mean ± SEM of quadruplicate values after subtraction of DFHBI-1T background and normalized for vehicle control.

The cell-free estrogen bioassay was validated by testing its capacity to detect estradiol in a dose-dependent manner (Figure 1). E2 was tested using 50% (Figure 2A) and 10% (Figure 2B) methanol:water (v/v) as vehicle. Data show that the higher methanol essentially blunted the estrogen activity response by ∼50% in comparison to 10% methanol. For 10% methanol, the assay showed 45% ER activation with 1362 pg/mL E2. It was important to establish a ∼50% activation for E2 as unknown eEDCs, especially in a mixture, may be more potent than E2 alone (for example through additive effects). Under these conditions, the bioassay exhibited a robust sensitivity with the limit of detection for E2 at 1.0 pg/mL. The EC_50_ for the E2 dose response at 10% methanol was 46.7 pg/mL (Figure 2B).

We next determined whether E2 spiked into 500 mL milliQ grade laboratory water could be detected in a dose-dependent manner. Following the spiking of water with E2 across the range 1.36–1362 pg/mL, the steroid was extracted using standard SPE columns. The eluate was dried to completion then reconstituted in 10% methanol:water (v/v) and tested in the cell-free estrogen bioassay. Water-extracted E2 could be readily detected and showed a strong dose-dependent effect (Figure 2C) demonstrating both the efficacy of the extraction process and the cell-free estrogen bioassay.

**Figure 3:**
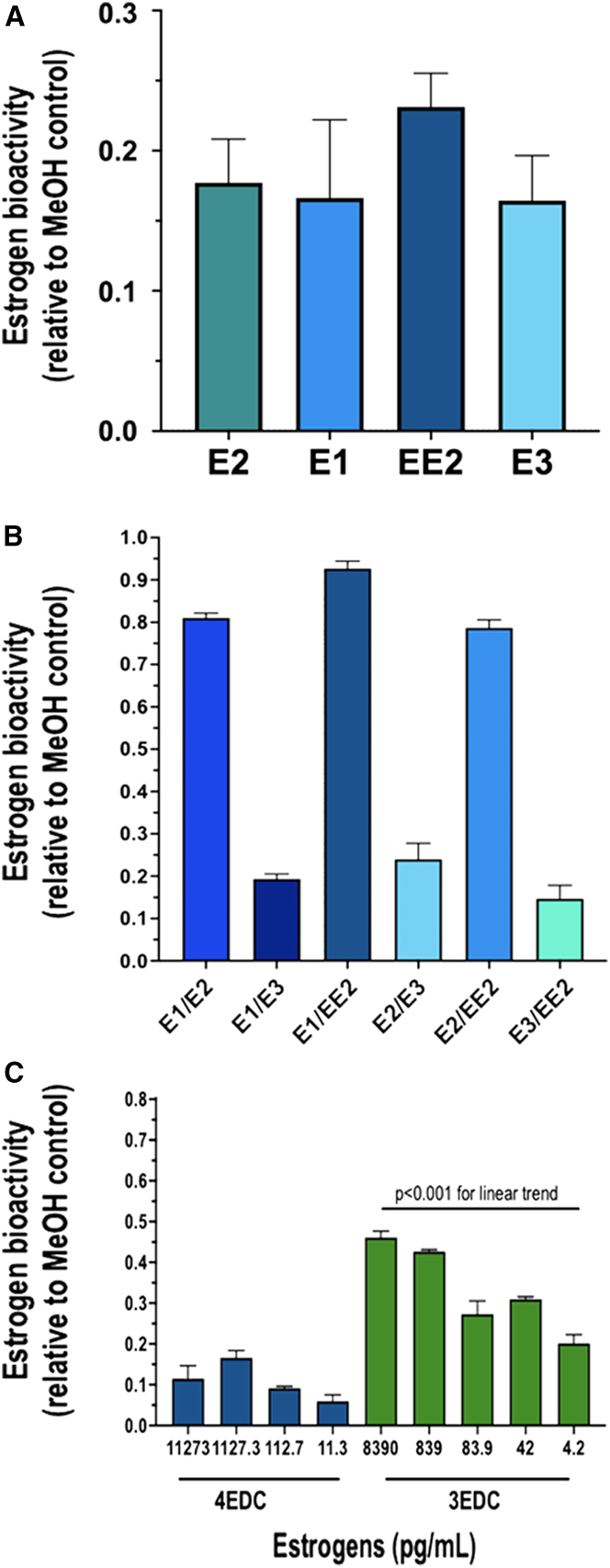
Estrogen receptor activation by single estrogens and combined mixtures (A) Estrogens (272 ng/L) 17β-estradiol (E2), estrone (E1), ethinylestradiol (EE2) and estriol (E3) diluted in 10% methanol were tested in the 1-h ER-IVT assay. (B) Pairs of the estrogens were tested, and data show the agonist effects of E1, E2, and EE2 with additive activity for all groupings (relative to single steroid in A), while E3 displays antagonistic activity in the presence of E1, E2, and EE2. (C) E1, E2, E3, and EE2 were mixed and spiked into 500 mL water (4EDC) or E1, E2, and EE2 were mixed and spiked into 500 mL water (3 EC) prior to SPE extraction. 4EDC showed no dose-response. 3EDC showed dose-response. All data are mean ± SEM of three biological replicate assays with four technical replicates each concentration or estrogen. For 3EDC, *p* < 0.001 using one-way ANOVA vs. blank control.

The next series of experiments demonstrated the ability of the cell-free estrogen bioassay to detect other estrogens. E2 was first tested at 13.5 pg/mL (lower concentration of the circulating levels of E2 in premenopausal females) and showed ER activity of 17.8%. Other endogenous hormones, estrone (E1, 13.6 pg/mL) and estriol (E3, 13.6 pg/mL) as well as ethinylestradiol (EE2, 13.6 pg/mL), the major synthetic estrogen in female contraceptives, were next tested and all showed a positive response with ER activities of 16.6%, 35.8%, and 30.2%, respectively (Figure 3A).

It is highly unlikely that any estrogenic molecule would be present in isolation in environmental samples and that it could contain any number of eEDCs. Therefore, to test if the cell-free estrogen bioassay could detect net estrogenic activity in the presence of multiple estrogenic molecules, we measured the net effect of combinations of E1, E2, E3, and EE2 (each added at 13.6 pg/mL). The net activities of E1/E2, E1/EE2, and E2/EE2 showed synergistic effect, with estrogenic activity of 80%–90% (Figure 3B) measured above that of E2 alone at 17.8% (Figure 3A). The net activities of E1, E2, and EE2 in combination with E3 were all reduced relative to other combinations without E3 (*p* < 0.001) (Figure 3B). This is in keeping with E3 being a relatively weaker estrogen than E2 and E1, and in the presence of E2 can diminish the biological responses mediated by E2 acting as a competitive inhibitor^21^^,^^22^

We then tested the combinations of E1/E2/EE2 and E1/E2/EE2/E3 for estrogenic activity when spiked into water to ensure the efficacy of our extraction and testing processes. In the presence of E1/E2/EE2, we observed a dose-dependent response in estrogenic activity as the concentrations of the spiked estrogens decreased across the total concentration range 8390 to 4.2 pg/mL (*p* < 0.001, Sidak post-hoc test) (Figure 3C). In the presence of the antagonist, E3, we could still detect net estrogenic activity, albeit at a lower level. The presence of E3 completely abolished the dose-dependent activity (Figure 3C). These data show that the cell-free estrogen bioassay can detect estrogenic activity in the presence of complex mixtures that include agonists or a combination of agonists and competitive inhibitors. The data also highlight that using dose-dependent dilutions of water extracts will provide some indication of whether agonists dominate a complex mixture or whether the complex mixture also contains competitive inhibitors.

### Detection of estrogenic activity in natural waterways

**Figure 4:**
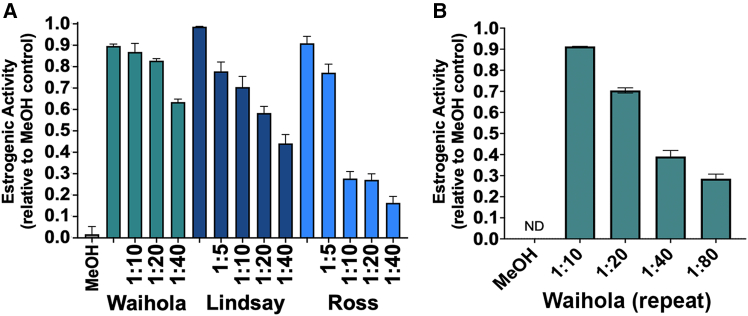
Relative ER activation by river and dilution (A) Relative ER activation measured in samples from Lake Waihola, Lindsay Creek, and Ross Creek across indicated dilutions. (B) ER activation measured in a repeat sample from Lake Waihola collected one month later. Data are represented as mean ± SEM from three biological assays, each analyzed using duplicates.

The cell-free estrogen bioassay was next used on real samples collected from natural waterways within, and surrounding Dunedin, New Zealand. For each sample, 500 mL water underwent SPE-extraction and the dried eluate resuspended in 100 μL 10% methanol:water (MeOH, Figures 4A and 4B). Samples from Lake Waihola, Lindsay Creek, and Ross Creek all showed strong estrogenic activity of 90%–95% for the undiluted extracted sample, with percentage estrogenic activity decreasing with increasing dilution (Figure 4A). The relative estrogen bioactivity values for these three waterways represents an E2 equivalence value of 369 000 ng/L, 61 ng/L, and 1.2 ng/L, respectively (Table 1). Given the high levels reported, Lake Waihola was retested approximately one month after the first sampling. Dose-dependent estrogen activity was again detected (Figure 4B). The dose-response curve varied between the two Waihola samples with the repeat sample showing 9-fold lower levels of estrogenic activity and E2 equivalence at 47,790 ng/L. Together, these data demonstrates that the cell-free estrogen bioassay can detect the presence of estrogenic compounds in natural waterways and that repeat sampling shows some variation that could be due to estrogen compound degradation and renewal or dilution and concentration effects due to weather patterns.

**Table 1:** E2 equivalence values for the waters tested in this study

Water source	E2 equiv (pg/mL)
Lake Waihola	69,197,833
Lindsay Creek	525,604,290
Ross Creek	91,491,670
Spring water 1	0
Spring Water 2	5501
Spring Water 3	2,263,293
Tap Water	18,115,955
Bottled Water A	0.08
Bottled Water B	164
Fountain Water 1	0
Fountain Water 2	483,970
Tank Water A	2,263,293
Tank Water B	3.7
Rainwater A	224,821,232
Rainwater B	37,295
Rainwater C	2072

To determine the potential compounds responsible for estrogenic activity in the waterways, samples from Lake Waihola, Lindsay Creek, and Ross Creek were screened using LC-HRMS (Tables 2 and 3). Each file was compared to a blank (100% methanol) and negative sample (extracted laboratory grade milliQ water eluted with 100% methanol) to exclude potential compounds that were from the preparation process. Table 4 shows the results from the LC-HRMS analysis with chemicals listed in descending order of abundance. Over 100,000 unique species were observed in chromatograms. Weak and poorly defined features (peak ratings <6.5) were excluded, along with features observed in blank injections. Tentative identities were proposed for the remaining features and included the known EDCs terbuthylazine, simazine, phthalic anhydride, EE2, methanedienone, and pregnenolone. Features were only named where there were good matches to exact mass and isotope distribution patterns for known compounds in databases. Confirmation of all species would require additional fragmentation pattern search and retention time comparison with a known standard.

**Table 2:** Optimised orbitrap mass spectrometry source and acquisition parameters used for EDC analysis

Parameter	Setting
Positive Ion (V)	3400
Negative Ion (V)	2000
Sheath Gas (Arb)	5
Sweep Gas (Arb)	5
ITT (°C)	350
Vaporiser (°C)	400
AGC Target	Standard

**Table 3:** Potential EDC analytes as standards at 1 ppm

Analyte	Accurate mass (Da)	Retention time (min)
Diethyl phthalate	222.0892	11.76
Dimethyl phthalate	194.1579	6.81
Di-*n*-butyl phthalate	278.1518	20.09
Di-2-ethylhexyl phthalate	390.2770	27.62
Methylparaben	152.0473	4.38
Propylparaben	180.0786	10.66
Ethylparaben	166.0630	7.38
Bisphenol A	228.1150	11.75
17β-Estradiol	272.1776	13.52

**Table 4:** Chemicals tentatively identified in water extracts by HRMS

m/z	Retention time (min)	Species	Tentative identification	Formula	Water sample (Area) #6	Water sample (Area) #9	Water sample (Area) #10
423.3308	20.93	[M + H]+	3,6,9,12,15,18-hexaooxactacosan-1-ol	C22H26O7	4.4e8	6.0e8	6.6e8
397.3324	28.06	[M−H]−	diisononyl adipate	C24H46O4	1.2e7	1.3e7	4.0e7
149.0231	19.98	[M + H]+	phthalic anhydride	C8H4O3	2.5e7	3.7e7	5.2e7
230.1165	13.75	[M + H]+	terbuthylazine	C9H16ClN5	3.1e6	5.4e5	3.0e7
369.3012	26.79	[M−H]−	bis(2-ethylhexyl)adipate	C22H42O4	1.2e7	1.5e7	2.7e7
348.2890	8.15	[M + H]+	anandamide	C22H37NO2	–	2.6e7	–
235.1690	13.94	[M + H]+	3,5-bis(*tert*-butyl)-2-hydroxybenzaldehyde	C15H22O2	2.5e7	9.0e6	–
317.2121	21.46	[M−H]−	steviol	C20H30O3	–	1.5e7	1.9e6
339.1221	13.14	[M + H]+	curcumin II	C20H18O5	1.5e7	4.0e5	–
301.2155	16.29	[M + H]+	methanedienone	C20H28O2	1.3e7	1.1e7	1.2e7
295.2163	5.43	[M + H]+	trimipramine	C20H26N2	1.3e7	4.0e6	3.3e5
427.3774	28.08	[M + H]+	diisooctyl sebacate	C26H50O4	2.6e6	3.1e6	9.8e6
295.1703	13.65	[M−H]−	ethinylestradiol	C20H24O2	–	9.8e6	–
355.1535	11.27	[M + H]+	xanthohumol	C21H22O5	8.2e6	–	–
341.1379	8.95	[M + H]+	(−)-8-prenylnaringenin	C20H20O5	7.0e6	–	–
471.3482	22.50	[M−H]−	maslinic acid	C30H48O4	6.0e6	4.9e6	8.2e5
202.0851	8.61	[M + H]+	simazine	C7H12ClN5	7.6e5	2.0e5	3.3e6
317.2469	25.31	[M + H]+	pregnenolone	C21H32O2	2.5e6	2.6e6	2.0e6

### Detection of estrogenic activity in natural and tap drinking waters

**Figure 5:**
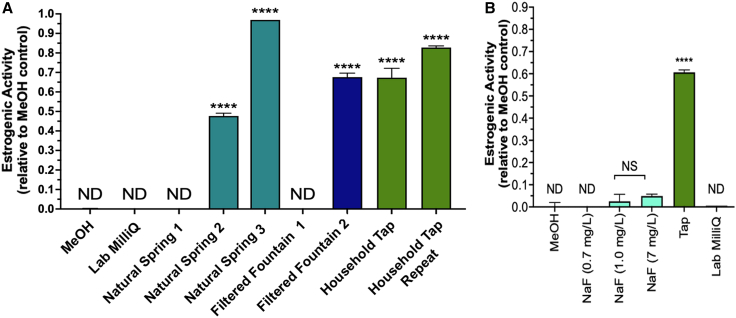
ER activation in natural, treated, and bottled water (A) Water samples from the laboratory (MeOH and MiliQ), natural spring 1 (Central Otago), natural spring 2 and 3 (in the Dunedin city area), filtered fountain 1 (University of Otago), filtered foundation 2 (gym location, Dunedin city area), household tap (Dunedin city area), household tap repeat one month after first sample. (B) Water spiked with vehicle (MeOH) or sodium fluoride (NaF) at 0.7 mg/L, 1.0 mg/L, or 7 mg/L, tap water and laboratory grade milliQ water. All data are presented as mean ± SEM for three biological assays with duplicate technical replicates. ND = estrogenic activity not-detected. ∗∗∗∗*p* < 0.001 by one-way ANOVA with Holm-Sidaks post-hoc tests vs. blank control. NS = not significant (*p* = 0.6380 1.0 mg/L NaF, *p* = 0.1943 7.0 mg/L NaF).

There have been an increasing number of reports describing the presence of eEDCs in drinking water.^23^^,^^24^ We next tested natural spring waters, filtered fountain waters available in a city gym and a university site, and household tap water. No estrogenic activity was detected in extracts of laboratory grade milliQ water, one source of spring water (fed by a snowy mountain range), and one of the filtered fountain water samples (Figure 5A). Significant estrogenic activity was identified in all the other samples including two spring outlets within Dunedin city, the gym-located filtered fountain water site, and household tap water (Figure 5A). To ensure that the latter observation was not the result of a single contaminating event, household tap water was retested approximately one month after the initial sample but found the same high level of estrogenic activity (Figure 5A). The estrogenic activity detected in tap water corresponds to an E2 equivalence of 96 ng/L (Table 1).

Dunedin household water is treated with fluoride as a preventative measure against dental disease, and previous studies have suggested that fluoride may interfere with estrogen signaling.^25^^,^^26^ Fluoride is added to Dunedin household water at a concentration of 0.7–1.0 mg/L and, as such, we tested the effect of 0.7–1.0 mg/L fluoride on activation in our ER-IVT assay. Laboratory grade MilliQ water spiked with 0.7 and 1.0 mg/L fluoride showed no significant increase in estrogenic activity (Figure 5B). Even at a concentration 10-times that used to treat Dunedin household water (7 mg/L) no significant increase in estrogenic activity was detected compared to the methanol control (Figure 5B). Together, the findings demonstrate that household tap drinking water has estrogenic activity that is not explained by fluoride treatment.

### Detection of estrogenic activity in rural areas

**Figure 6:**
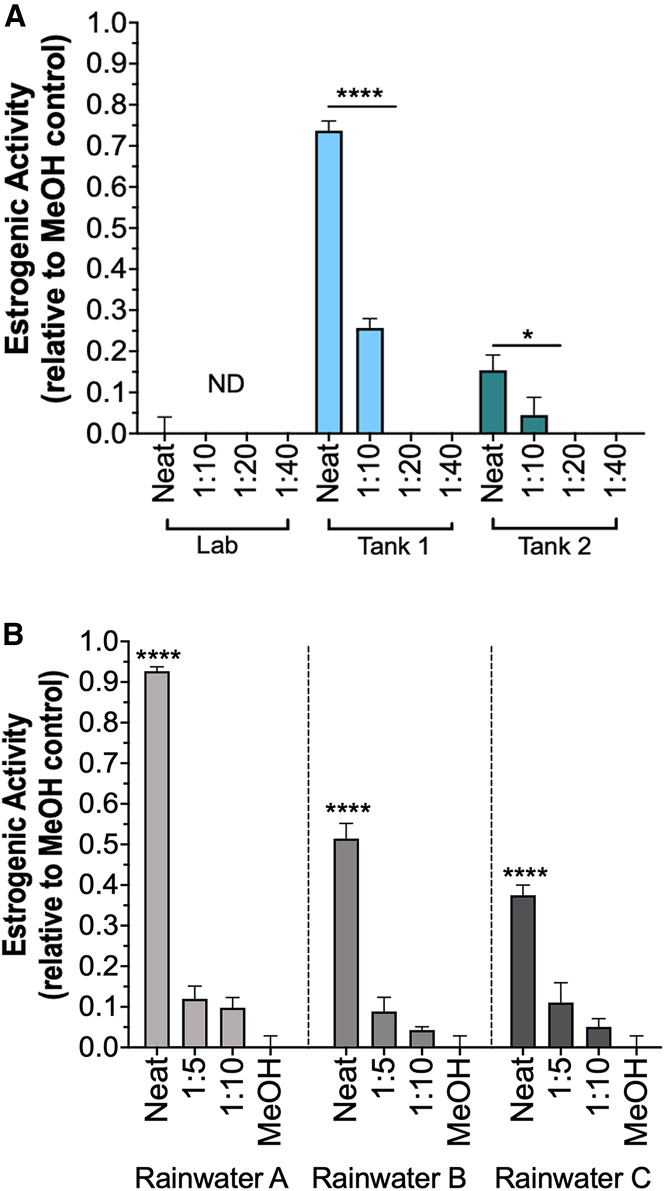
ER Response in harvested rainwater and storage tanks (A) Water samples from storage tanks near airport (1) and near coast (2). (B) Water collected during rainfall events over a single weekend from different areas (A, 400 mL; B, 200 mL) in the city or over a different weekend (C, 150 mL, same site as A). The rainwater extracts were compared to a methanol-spiked laboratory water control (MeOH). All data are mean ± SEM from three biological assays, each with duplicate technical replicates. ND = estrogenic activity not-detected, ∗∗∗∗*p* < 0.001 by one-way ANOVA with Holm-Sidaks post-hoc test vs. blank control (MeOH).

The areas surrounding Dunedin city are largely rural areas where water for farming use is collected in large polymer tanks. We collected water samples from two areas, one located close to the Dunedin airport (Tank A) and the other nearer the coast (Tank B). Both tank water samples tested positive for estrogenic activity, albeit at different levels with the tank close the airport showing 75% estrogen activity, correlating with an E2 equivalence of 47,908 ng/L (Figure 6A; Table 1). The coastal region showed much lower estrogen activity at just 15%, and E2 equivalence of 0.097 ng/L.

### Detection of estrogenic activity in rainfall

We next collected rainfall water across a 24-h period at three locations across Dunedin city. At the different sites, different volumes were collected, with 400 mL for site A, 200 mL for site B, and ∼150 mL for site C. Each site showed estrogenic activity, with the different estrogenic activity reflective of the volume collected (Figure 6B). Correcting for the different dilution factors associated with volume collected, the rain waters had E2 equivalence concentrations of 0.22 ng/mL, 37 ng/mL, or 2 ng/mL, respectively. The complexity of eEDCs appears to be different from the natural riverways, or tap water, with the dilution series decreasing much more rapidly for rainwater versus Lake Waihola (Figure 4), for example.

### Detection of estrogenic activity in bottled water

**Figure 7:**
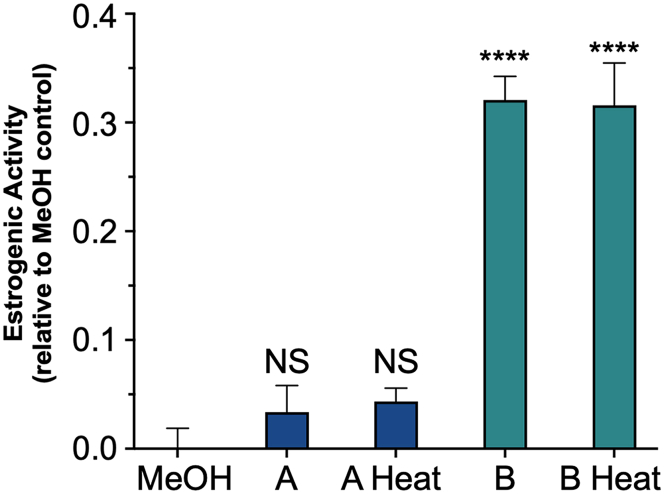
Estrogenic activity across commercial bottled water products Relative ER activation measured in vehicle control (MeOH), sample A, sample A following heat treatment, sample B, and sample B following heat treatment. A-high-end commercial brand, B-low-end commercial brand. Data are presented as mean ± SEM (*n* = 3 biological replicates; 2 technical replicates per sample). NS = not significant, ∗∗∗∗*p* < 0.001 by one-way ANOVA with Holm-Sidaks post-hoc test vs. blank control (MeOH).

Plastic wrappings and containers have long been reported to contain compounds that can leach into foods and water. Many of these compounds such as BPA and phthalates have well established estrogenic activity.^27^^,^^28^ Therefore, we next tested two brands of bottled sparkling water for estrogenic activity. We purposefully chose a high-end brand versus a low-end brand to compare estrogenic activity present in the water. Water bottles can be exposed to extreme temperatures, for example, left in a car during summer, so we also tested the water in these bottles after they had been subjected to 40°C for 8 h. The high-end water (Brand A) did not contain estrogenic activity under any conditions (Figure 7). In striking contrast, significant estrogenic activity was detected in Brand B, with no difference upon heating (Figure 7).

## Discussion

Using synthetic biology, we have developed a new ER-IVT reporter gene assay that enables any estrogenic compound activating the ER to be detected with high sensitivity. The assay has a detection limit of 1.0 pg/mL for E2 but has been designed so that a wide range of eEDC potencies above and below that of E2 itself can be detected. This is especially important as eEDCs in the environment most likely occur in combination. Using this methodology, we demonstrate here that estrogenic activity is present in many, but not all, water sources, including natural riverways, and drinking water sourced from natural springs or household tap supply, as well as a commercially available bottled source. The detection of estrogenic activity in a diverse range of water sources raises both human and ecological health concerns.

The newly developed ER-IVT assay is designed as an inhibition assay, capitalizing on the fact that estrogen activation of the estrogen receptor (ER) suppresses T7 RNA polymerase activity. This inhibitory format offers a distinct advantage: it enables the detection of very low concentrations of estrogens, with limits of detection in the pg/mL range. Such sensitivity rivals or even surpasses many established cell-based assays, including K47D-KBluc (human breast cancer line, luciferase under ERE control; LOD 1–3 pg/mL) and the E-Screen (MCF-7 proliferation assay; LOD 3–6 pg/mL). While CALUX (Chemically Activated LUciferase gene eXpression) remains the benchmark for sensitivity (LOD ∼0.3 pg/mL), the cell-free ER-IVT system offers a unique flexibility. Because the assay is built on a defined *in vitro* transcription system, each component can be precisely titrated to fine-tune performance, opening the potential to drive sensitivity even lower if desired. In its current configuration, the assay has been specifically optimized for detecting trace levels of eEDCs, making it a powerful and adaptable alternative to traditional cell-based assays.

The successful application of the novel ER-IVT reporter assay on extracts from river and lake samples revealed significant estrogenic activity in all three rivers tested. The enduring nature of the estrogenic activity was further confirmed by repeat sampling, which showed that estrogenic activity persists, at least at one site. This finding is consistent with what has been found for other countries where eEDCs have been detected in natural waterways.^2^^,^^3^^,^^29^^,^^30^^,^^31^^,^^32^^,^^33^ These pollutants can enter surface and groundwater through domestic sewage, agricultural run-off and effluent from industrial and wastewater plants.^34^ eEDCs are difficult to remove from sewage and wastewater effluents^35^^,^^36^^,^^37^ and given the lack of any consensus approach, removal efficiency can vary from zero to 99%.^38^

Steroid hormone use in pharmaceuticals, livestock, and animal husbandry significantly contributes to water contamination. It is estimated that the human population excretes approximately 30,700 kg of natural and synthetic estrogens per year. However, this only accounts for 37% of these compounds as livestock in the USA and EU contribute another 83,000 kg/year.^39^^,^^40^ Natural and synthetic estrogens are not the only source of the estrogenic activity with chemicals such as alkyl phenols, bisphenols, and phthalates^41^ all able to leach out of plastics or enter surface waters through use of fertilizers, car oils, detergents, shampoos, and lotions. Hence, it is likely that the net estrogenic activity measured in waterways represents a combination of natural and synthetic estrogens together with chemicals known to have estrogenic activity. Indeed, we show here by LC-HRMS the presence of such a variable grouping with pregnenolone, ethinylestradiol, phthalate anhydride, terbutazine, simzanone, and several phytoestrogens all identified in the waterways we tested.

An interesting finding from this study was the detection of eEDCs in outside tank water. The primary source of the tank water was rainfall, which suggests that the rainwater itself contained eEDCs. To investigate this, we collected rainwater at two different sites on the same day and repeated the process at a third site during a separate rainfall event. Using the ER-IVT reporter assay, we were able to clearly demonstrate the presence of estrogenic activity in the rainwater samples. This finding aligns with earlier studies, where a range of eEDCs, including BPA, alkylphenols, phthalates, and flame retardants, were found in rainwater collected from multiple locations in the Netherlands.^42^ In China, chemical analysis of rainwater runoff from roofs identified 54 contaminants, 30% of which were classified as EDCs.^43^ Some studies have indicated that atmospheric deposition in areas of industrial activity or agricultural practices is a significant source of EDCs, including the eEDCs BPA and phthalates, suggesting that these chemicals can be transported via the atmosphere and deposited into rainwater.^44^^,^^45^

At present, testing for eEDCs in water is focused on the targeted detection of a few chemicals. Typically, BPA, certain phthalates, E2, E1, EE2, and pesticides are monitored and for each of these, there are specific and sensitive tests available. However, eEDCs do not exist in isolation, and with thousands of organic compounds now identified as potential eEDCs, it is likely that most samples contain a wide mixture of these compounds. As such, current detection practices may greatly underestimate the estrogenic threat present in any one sample. The use of an *in vitro* bioassay helps overcome some existing limitations of compound-specific monitoring by providing a functional measure of net estrogenic activity in a sample. This approach allows the activity to be expressed as E2 equivalence (E2_eq_) and we find this to range from 1.2–3.7 × 10^5^ ng/L in the natural waters tested here. By comparison, the levels of BPA alone in the Yellow river in China were reported as being in the middle of this range at 2.75 × 10^4^ ng/L.^46^

The levels of estrogenic activity measured in the natural waters would be expected to impact ecological health with aquatic species, such as fish and amphibians, being particularly vulnerable to eEDCs. Studies have shown that exposure of these organisms to eEDCs in the pg/L range can lead to feminization, hermaphroditism, males with extra testes, discontinuous gonads, all of which results in disrupted reproductive behavior.^47^^,^^48^^,^^49^^,^^50^ We detected eEDC activity in natural waters where aquatic species are found, and for at least one site, the eEDC activity was repeat measured one month apart, showing the likely continuous exposure of aquatic species to these estrogenic contaminants. Together with the known bioaccumulation of eEDCs in tissues, it follows that eEDCs over time could threaten ecosystem health and potentially destabilize entire ecological communities.^51^^,^^52^

Human sources of drinking water including natural, treated, and bottled varieties also showed estrogenic activity. While one natural spring water, one filtered water and one bottled source was free of estrogenic activity, all other sources tested showed estrogenic activity. The presence of eEDCs likely stems from the contamination of the drinking water source by surface and groundwaters after the failure of sewage treatment to eliminate estrogenic compounds. Our findings for New Zealand align with the reported contamination of drinking water in other countries by the natural and synthetic hormones E2, E1, and EE2 the plasticizers, BPA and diethyl phthalate, and the fertilizers, simazine and terbuthylazine. Concentrations of plasticizers can be up to 6.7 × 10^6^ ng/L while lower levels of E2, EE2, and E1 and fertilizers have been reported (up to 2.4- and 4.1 ng/L, respectively).^53^^,^^54^^,^^55^ We find here that the net E2_eq_ of household tap water is 96 ng/L.

The key question is whether the E2_eq_ contamination measured in the drinking waters is relevant to human health. Although difficult to establish, it is possible that ingesting this level of E2_eq_ may contribute to the estrogenic *milieu* of an individual. The recommended water intake per adult is a minimum of 2L per day, indicating that 192 ng E2_eq_ could be ingested. Not all ingested E2_equiv_ will reach circulation due to adsorption and liver metabolism and actual bioavailability has been calculated to range from 0.1% for compounds such as BPA to 55% for EE2.^56^ As such, we estimate that 19.2–105.6 ng of ingested E2_eq_ could reach the bloodstream resulting in additional 3.8–21.1 ng/L estrogenic activity. Given that the concentrations of circulating natural E2 in women fluctuate from 100 to 800 ng/L across the menstrual cycle,^57^ it is likely that ingested eEDCs will not immediately effect natural estrogen signaling. However, eEDCs are known to accumulate in lipids providing an on-going reservoir of estrogenic activity regardless of current exposure.^58^^,^^59^ Together, this raises the possibility that eEDCs from contaminated water sources may exert persistent biologically meaningful effects across the normal human lifespan.^60^

Most studies examining the estrogenic effects of eEDCs on health have concentrated on BPA. Used at equimolar concentrations to E2 (10^−12^ M), BPA is known to produce a transcriptional signature profile in MCF-7 cells that overlaps with E2.^61^^,^^62^ Recent studies in rodents using orally administered BPA (0.25 μg/kg/day or 5136 ng) have demonstrated a wide array of adverse *in vivo* effects. In terms of breast cancer alone, these include the development of preneoplastic^63^^,^^64^ and neoplastic lesions^65^ in the mammary gland *in utero*, increased susceptibility to mammary gland tumors in the perinatal period, and the acceleration of mammary tumors in tumor-prone mice, such as those carrying BRCA1 mutations^63^^,^^66^^,^^67^ in adult mice. The earlier claims that eEDCs were “weak estrogens” because 10,000–100,000 higher doses are required to induce certain cellular endpoints^68^^,^^69^ has been challenged by the recognition that initial cell culture approaches were unlikely to be replicating *in vivo* conditions.

In humans, multiple studies have highlighted the association of eEDCs with reproductive health concerns in men and both pre- and postmenopausal women. These include reduced sperm counts, earlier onset of puberty as well as menopause, irregular menstrual cycles, and infertility.^70^^,^^71^ Furthermore, eEDCs can exacerbate other endocrine disorders that directly or indirectly impact uterine health, including PCOS, endometriosis, endometrial cancer, and ovarian cancer.^72^^,^^73^ There is little doubt that humans are exposed to eEDCs as they are detected in multiple human tissues,^74^^,^^75^ including adult and fetal serum where they occur in a typically complex mixture.^76^^,^^77^

In summary, we report here the use of synthetic biology to develop a novel cell-free IVT assay that allows the functional estrogenic activity of water samples to be measured with relative ease. We show that significant estrogenic contamination exists in multiple water sources in the Dunedin city of New Zealand. With a detection limit of 1 pg/mL for E2, the assay is shown to be able to detect multiple estrogenic compounds including E1, E2, E3, and EE2. We detected estrogenic activity in some, but not all, waterways, spring waters, tap water supplies, and bottled water sources. Perhaps the most surprising was the detection of estrogenic activity in tank and rainwater, suggesting atmospheric contamination from vehicle exhaust, industrial emissions, or agricultural practices. Overall, this study indicates the usefulness of IVT bioassays in screening for functional estrogenic activity resulting from essentially any single or group of compounds that can activate the ER. In conjunction with subsequent directed MS analysis, this technology is likely to provide a significant boost to the worldwide detection and monitoring of eEDCs in our increasingly contaminated waterways.

### Limitations of the study

This study has several limitations. The study measures ER bioactivity, as a proxy for estrogenic EDCs, however, only measures agonistic activity and does not measure antagonistic compounds in this experimental setup. EDCs encompass many more compounds other than disruption of estrogen signaling, so these results are likely an underestimate of the full disruption. We only used one extraction process which likely produced variable recovery between different classes of EDCs so may have introduced over- or under-estimation. ER bioactivity measurements in tank/rainwater suggests atmospheric origin, however, without air sampling for estrogenic EDCs, the data for these environmental samples are hypothesis-generating at best, not causal-proof. Our EDC data implies ecological and human health relevance; however, we did not directly assess within this study.

## Resource availability

### Lead contact

Requests for further information and resources should be directed to and will be fulfilled by the lead contact, Professor Alison K. Heather, alison.heather@otago.ac.nz.

### Materials availability

There are restrictions to the availability of the IVT reaction because of intellectual property protection. As such, reagents generated in this study will be made available on request, but we may require a payment and/or a completed material transfer or license agreement if there is potential for commercial application.

### Data and code availability

• Data will be made available upon reasonable request.
• This paper does not report original code. Any additional information required to reanalyze the data reported in this paper will be made available from the lead contact upon reasonable request.

## Acknowledgments

This work was funded by InsituGen Ltd, Dunedin, Otago, New Zealand.

## Author contributions

G.M. contributed to water sampling, experimental work and design, data analysis, manuscript writing, and revisions. A.G., E.S.S., and C.M.D. participated in experimental work, data analysis, and manuscript revisions, with C.M.D. and E.S.S. also contributing to water sampling. K.L.S. was involved in experimental work and design, data analysis, manuscript writing, and revisions. A.K. contributed to experimental work and design, data analysis, water sampling, manuscript writing and revisions, and laboratory management. A.K.H. was responsible for the experimental conception and design, data analysis, manuscript writing, final manuscript revision, and overall project oversight.

## Declaration of interests

A.K.H. is an employee and shareholder of InsituGen Ltd (NZ). G.M., A.G., E.S.S., C.M.D., and A.K. are all employees of InsituGen Ltd (NZ). There is a patent application for the novel assay disclosed in this research work. International PCT Application No: PCT/NZ2020/050046 “Novel Ligand Assays”.

## STAR★Methods

### Key resources table

**Table:** 

REAGENT or RESOURCE	SOURCE	IDENTIFIER
**Biological samples**		

Rainwater	South Island, NZ	
Water Ways	South Island, NZ	
Spring Waters	South Island, NZ	
Tap Water	Dunedin City, South Island, NZ	
Bottled Water	Supermarkets, Dunedin City, NZ	

**Chemicals, peptides, and recombinant proteins**		

RPMI 1640 culture medium	Gibco	11875093
10% Hyclone Fetal Bovine Serum	GE Life Sciences	SH30406.02
phosphate buffered saline	AppliChem	A09659010
cOmplete EDTA free protease inhibitor	Roche	04693132001
PhosStop phosphatase inhibitor	Roche	04906837001
PMSF	Sigma-Aldrich	93482-50 ML-F
RIPA Lysis Buffer	Sigma-Aldrich	RO278-50 ML
Filter Paper MGC Dia 47 mm Glass Fiber	Munktell Ahlstrom	3.1103.047
Membrane Filter Cellulose Acetate 0.20um NS	Munktell Ahlstrom	760107
TrypLe Express	Gibco	12604021
Potassium Phosphate, Monobasic	Merck	529568-250 GM
Sodium Chloride	Merck	1.06404.1000
Triton X-100 solution	Sigma-Aldrich	93443-100 mL
DTT for molecular biology 5g	neoFroxx	1114GR005
D-(+)-Trehalose Solution 500 mL, 1M	Life Sciences Technologies Inc.	TS1M-500
Oasis HLB 6 cc Vac Cartridge, 200 mg Sorbent per Cartridge, 30 μm	Waters	WAT106202
Methanol for analysis EMSURE ACS, ISO, Reag. Ph Eur	Merck	1060092500
Restriction Enzyme SnaBI	New England Biolabs	R0130M
Milli-Q water	Millipore/Sigma	Millipore Milli-Q gradient
NTPs	New England Biolabs	N2052AVIAL
HSP90 beta protein	Abcam	ab 80033
RNaseI inhibitor	New England Biolabs	M0314L
T7 RNA polymerase in trehalose	Life Biosciences Inc.	T7T-70000
17β-estradiol	Merck	3301
Estrone	Merck	E9750
Ethinylestradiol	Merck	PHR1480
Estriol	Merck	PHR2800
Na_2_HPO_4_	Merck	1065800500
KCl	Merck	1049360500
1M MgCl_2_	Thermo Fisher	AM9530G
DFHBI-1T Fluorophore 10 mg	Tocris	561010
Dimethyl Sulfoxide DMSO 100 mL	Calbiochem/Sigma	317275-100 ML
POLOXAMER 188, 10% SOLUTION	Thermo Fisher	24040032

**Critical commercial assays**		

PureLink HiPure Plasmid Maxiprep kit	Thermo Fisher Scientific/Invitrogen	K210007
GenepHLow DNA Cleanup Midi kit	Geneaid	DFI 100

**Experimental models: Cell lines**		

Estrogen Receptor Luciferase Reporter T47D Stable cell line	Signosis, Santa Clara, CA, USA	SL-0002

**Recombinant DNA**		

pIDTSMART-AMP+		

**Other**		

Filtration Set 47 mm VF6 Glass	Rocker Scientific	167200–06
Concentrator Speed Vac	Eppendorf	5305000380
FLUOstar Omega	BMG Labtech	
Spectramax i3X	Molecular Devices	
Thermo Exploris 480 Orbitrap Mass Spectrometer (LC-HRMS). Liquid Chromatography (LC) separation was performed on an Acquity UPLC BEH C18 column		

### Method details

#### Processing and extraction of water samples

The protocol used in this study was adapted from the following source.^78^ Briefly, glass bottles were autoclaved before sample collection. For all water samples, 0.5-1.0 L was collected, unless otherwise stated. Once collected, the bottles were stored in the dark at 4°C for up to 48 hours. For rainwaters, a Milli-Q grade washed laboratory beaker was left out overnight during a period of rain. To begin the extraction, 500 mL was filtered twice through a 47 mm diameter glass fiber membrane (3.1103.047, Munktell Ahlstrom) and then filtered through a 0.20 μm cellulose acetate membrane (760107, Munktell Ahlstrom) using the filtration system (Rocker, ROC 167200-06). The filtrate was then applied to a pre-conditioned Waters Oasis HLB column (Waters; WAT106202). Pre-conditioning involved flushing 5 mL of methanol (MeOH) and then 5 mL of Milli-Q grade laboratory water through the column. The sample was then applied to the column at a flow rate of ∼10 mL/min under vacuum 2-7 Hg. Bound steroids were eluted into 15 mL collection tubes with 5 mL MeOH. The eluate was then dried to completion (approximately 1.5 hours) in a concentration speed vac (Eppendorf EPP5305000380) at 60°C. The dried extracts were reconstituted in 100 μL methanol:nuclease free water (1:1) and stored at -80°C until tested for estrogenic activity. Negative controls for the experimental protocols consisted of methanol spiked into laboratory grade MilliQ-MilliPore (Sigma-Aldrich) Type 1 water and solid phase-extracted as above.

#### Preparation of the DNA template

The DNA template was commercially synthesized (Integrated DNA Technologies, Inc.) and cloned into plasmid pIDTSMART-AMP+. Once cloned, the plasmid was transformed into *E.coli* for amplification, transformants cultured and the plasmid DNA purified using PureLink HiPure Plasmid Maxiprep kit (Thermo Fisher Scientific/Invitrogen K210007). For use in the IVT reactions, the plasmid was linearized with SnaBI (New England Biolabs, R0130M) in a 200 μL reaction mixture that included 20U of enzyme per 20 μg DNA and was incubated at 37°C for 2 hours. Linearized DNA was purified using GenepHLow DNA Cleanup Midi kit (Geneaid DFI 100) and stored at -20°C before use. Linearization of plasmid DNA was checked using agarose gel electrophoresis.

#### Preparation of estrogen receptor (ER) lysate

The Estrogen Receptor Luciferase Reporter T47D Stable Cell Line (Signosis, Santa Clara, CA, USA, SL-0002) was cultured in RPMI 1640 culture medium (Gibco, 11875093) supplemented with 10% Hyclone Fetal Bovine Serum (GE Life Sciences SH30406.02) and 1% (v/v) penicillin/streptomycin at 37°C with 5% CO_2_. Media was aspirated and cells were washed twice with 1X phosphate buffered saline (PBS 10X Dulbecco, AppliChem A09659010) before cells were harvested with the addition of TrypLe Express cell dissociation solution (Gibco, 12604021). Detached cells were centrifuged gently (300xg, 5 mins) and the pellet was resuspended in 1XPBS. Cell number was then determined before 6-8X10^7^ cells/mL were re-centrifuged (100xg, 5 mins at 4°C) and the pellet resuspended in 5 mL ice-cold RIPA lysis buffer supplemented with cOmplete protease inhibitor solution (Roche 04693132001), PhosStop phosphatase inhibitor (Roche 4906837001), and PMSF (Merck, 93482) and incubated on ice for 30 mins. Lysates were then vortexed and centrifuged at 21,000xg for 20 mins at 4°C. The protein concentration of the supernatant was determined by spectrophotometry (A_230_ nm). Lysate was diluted in 146 mM trehalose buffer (20 mM potassium phosphate pH 7.5, 100 mM NaCl, 5 mM DTT, 0.2% Triton X-100, 146 mM trehalose) to a working solution of 25 ng/μL and stored at -80°C. The cell lysate preparation provides enough reagent for > 1 million reactions so the same lysate preparation was used for all experiments reported in this study.

#### IVT reaction

IVT reactions comprised ice-cold nuclease-free water to a final volume of 20 μL, 3.75 μL transcription buffer containing NTPs (NEB N2052AVIAL), 50 ng linearized DNA template, 90 ng heat shock protein 90 (HSP90, Abcam ab 80033), 90 ng ER lysate in trehalose buffer, 20U RNaseI inhibitor (NEB M0314L), and 15U T7 RNA polymerase (Life Biosciences T7T-70000) and 1 μL of eluate or 17β-estradiol (E2, Merck 3301), estrone (E1 Merck E9750), ethinylestradiol (EE2, Merck PHR1480), or estriol (E3, Merck PHR2800). Methanol 0.1 % (v/v) was included as control for all experiments. Reaction tubes were incubated at 37°C for 60 mins then immediately placed on ice and mixed with 80 μL detection buffer [10 mM Na_2_HPO_4_, 125 mM KCl, 5 mM MgCl_2_, 1% Poloaxmer 188, pH 7.5, 2 μL of 1 mM DFHBI-1T (Tocris, 561010)]. The fluorescence of iSpinach-activated DFHBI-1T was measured using a FLUOstar Omega (BMG Labtech) or Spectramax i3X (Molecular Devices) plate reader with excitation 460 nm and emission 505 nm.

For E2 dose response curves, the steroid was diluted in 50% or 10% methanol:water (v/v) across the range 1.36 pg/mL to 1362 pg/mL, covering the physiological range of circulating E2 in young premenopausal females.

#### High resolution mass spectrometry analysis

EDC separation was performed with an Ultimate 3000 HPLC coupled to a Thermo Exploris 480 Orbitrap Mass Spectrometer (LC-HRMS). Liquid Chromatography (LC) separation was performed on an Acquity UPLC BEH C18 column (100 x 2.1 mm ID, 1.7 μm, Waters) at 55°C in a linear gradient (20-95% over 29 minutes) with water and methanol as the mobile phases, both containing 0.1% formic acid. The mass spectrometer was equipped with an electron spray ionization source (H-ESI) operated in positive and negative mode with parameters described in Table 2. Data were acquired over the mass range of 100-1000 Da using full scan MS mode at 120,000 resolution in conjunction with an internal mass calibration (EASY-IC). This allowed a non-targeted approach to the analysis. Sensitivity of likely target analytes (Table 3) were checked to ensure optimal ionisation and separation parameters.

Water extracts were transferred to a HPLC vial and 10 μL injected into the LC-HRMS. Data generated from the LC-HRMS were processed using the Compound Discoverer 3.3 SP2 application (Thermo Scientific Fisher). Retention times were automatically aligned, and peak assignment was performed with a mass tolerance of 5 ppm and retention time tolerance of 0.1 min. The workflow searched for preferred adducts (M+H, M-H) as well as isotope pattern detection (Br;Cl). Identification was performed by a local database and other data sources such as MassList, Chemspider and mzCloud.

### Quantification and statistical analysis

For each experiment, the data is presented as the mean ± SEM. At least 3 independent experiments on three different days were performed on each water sample. All reactions were performed in duplicate or triplicate. The methanol control was used as the baseline, representing 0% estrogenic activity, and all experimental results were normalized to this control in each assay. Statistical significance was assessed using one-way ANOVA, followed by appropriate post-hoc tests to determine differences between groups. The specific post-hoc test applied for each experiment is detailed in the respective Figure Legends.

To calculate E2 equivalence (E2_eq_) for the estrogenic activity of the water samples, the E2 dose response curve was analyzed using a logistic regression model to generate a nonlinear sigmoidal curve (4PL model) (Figure 2C). The 4PL equation of Y = [(A1-A2)/1+(X/EC_50_)p] +A2 was then solved for X for each sample (Y=estrogenic bioactivity).
